# Estimation of diffusion constants from single molecular measurement without explicit tracking

**DOI:** 10.1186/s12918-018-0526-5

**Published:** 2018-04-11

**Authors:** Shunsuke Teraguchi, Yutaro Kumagai

**Affiliations:** 1 Tohoku Medical Megabank Organization, Tohoku University, 2-1 Seiryo-machi, Aoba-ku, Sendai, 980-8573 Japan; 2 Quantitative Immunology Research Unit, Immunology Frontier Research Center, Osaka University, 3-1 Yamada-oka, Suita, Osaka, 565-0871 Japan

**Keywords:** Brownian motion, Diffusion constants, Expectation maximization algorithm, Probabilistic model, Single molecular measurement

## Abstract

**Background:**

Time course measurement of single molecules on a cell surface provides detailed information about the dynamics of the molecules that would otherwise be inaccessible. To extract the quantitative information, single particle tracking (SPT) is typically performed. However, trajectories extracted by SPT inevitably have linking errors when the diffusion speed of single molecules is high compared to the scale of the particle density.

**Methods:**

To circumvent this problem, we develop an algorithm to estimate diffusion constants without relying on SPT. The proposed algorithm is based on a probabilistic model of the distance to the nearest point in subsequent frames. This probabilistic model generalizes the model of single particle Brownian motion under an isolated environment into the one surrounded by indistinguishable multiple particles, with a mean field approximation.

**Results:**

We demonstrate that the proposed algorithm provides reasonable estimation of diffusion constants, even when other methods suffer due to high particle density or inhomogeneous particle distribution. In addition, our algorithm can be used for visualization of time course data from single molecular measurements.

**Conclusions:**

The proposed algorithm based on the probabilistic model of indistinguishable Brownian particles provide accurate estimation of diffusion constants even in the regime where the traditional SPT methods underestimate them due to linking errors.

**Electronic supplementary material:**

The online version of this article (10.1186/s12918-018-0526-5) contains supplementary material, which is available to authorized users.

## Background

Sensing the extracellular environment is crucial for cells to properly respond and function. The information from the environment is typically encoded in microscopic molecular signals that are recognized by cell surface receptors. The signaling of cell surface receptors involves several physical processes, including ligation to their ligands, oligomerization, and subsequent binding to the downstream signaling components in cytosol. Although many details of these processes have been inferred from biochemical, genetic, and molecular or cell biological studies, their physical and dynamical aspects at the microscopic level are still largely unknown [1].

Recent development of techniques for single molecular measurement such as total internal reflection fluorescence (TIRF) microscopy [2] provides a chance to directly observe the dynamics of these processes from time course images of fluorescently-labeled single molecules on cell surfaces [3, 4]. A typical workflow for such data is single particle tracking (SPT) [5]. In SPT, the positions of particles in each time frame are first detected. With the help of the sophisticated detection algorithms, the spatial resolution of the detected position could be of sub-pixel order [6]. The next step is linking, where the trajectory of each molecule is inferred by connecting seemingly identical particles in subsequent frames. Usually, the nearest particle in the subsequent frame with global consistency is identified as the same particle [7, 8].

The identified trajectories of particles must be further analyzed quantitatively to find biologically relevant physical parameters. The diffusion constant, which characterizes the diffusion speed of the particles, is one such parameter, and has been the target for subsequent analyses [9–11]. It has been shown that the diffusion constants of membrane proteins such as cell surface receptors can change along with biophysical events such as binding to their ligand or cytosolic adaptor molecules. For example, the diffusion constants of the epidermal growth factor receptor (EGFR), which belongs to a family of receptor tyrosine kinase, have been found to decrease after binding to EGF, and to transduce signals via subsequent binding with its adaptor Grb2 protein [12, 13]. It has also been shown that intracellular signaling proteins functioning on the membrane have multiple states, each of which have different diffusion constants [14, 15].

Although SPT methods are widely used, they encounter difficulties when the density of particles is higher. When the particle density becomes comparable to the scale of diffusion in the time resolution of the measurement, the expected area of diffusion of a particle tends to contain several irrelevant particles purely by chance. Since, in typical experiments, visualized molecules are indistinguishable from fluorescent signals, linking errors of SPT are inevitable. Then, trajectories from such erroneous SPT lead to underestimation of diffusion constants, and incorrect biological interpretations. Note that this problem of linking error may occur even in the regime where the detection error coming from the diffraction limit of a microscope is negligible.

In this paper, we address this problem of linking error in diffusion constant estimation. As we have seen, the problem arises from the impossibility of perfect hard linking of identical particles in SPT. Here instead of linking the nearest particles in subsequent frames, we only assign a probability of such possible identification with respect to the particle density around the position, and directly estimate the diffusion constant without specifying concrete trajectories. For this purpose, we derive a probabilistic model of the distance to the nearest neighbor by generalizing the canonical theory of single Brownian motion into multiple indistinguishable particles. The resultant algorithm successfully estimates diffusion constants even under high particle density conditions where SPT based methods underestimate them. The proposed algorithm shows some resemblance to another SPT free diffusion constant estimation method, namely particle image correlation spectroscopy (PICS) [16], which was inspired by image correlation microscopy [17–20]. The advantages of our algorithm over PICS include lower variances of estimated diffusion constants, lower numbers of hyperparameters to be determined before the analysis, and the applicability to cases with inhomogeneous particle distributions, whereas PICS assumes a homogeneous distribution.

In this paper, we first introduce the probabilistic model of the positions of the nearest neighbors of a diffusing particle surrounded by indistinguishable particles and then formulate the inference of diffusion constants in terms of maximum likelihood estimation based on this model. In a simple setting with a homogeneous particle distribution, our algorithm can be considered to be a natural generalization of the canonical diffusion constant estimation from the mean square displacement (MSD) to the case of finite density of surrounding particles. Our algorithm is further generalized to allow multiple states with different diffusion constants with the help of the expectation maximization (EM) algorithm [21]. Comparison of the performance of our proposed method based on simulated artificial diffusion data with other diffusion constant-estimation methods indicates the advantage of the proposed algorithm. Finally, we demonstrate that the algorithm can be used to infer the state of each molecule and visualize the single molecular data with such information.

## Theory

### A probabilistic model of a diffusing particle surrounded by indistinguishable particles

**Fig. 1:**
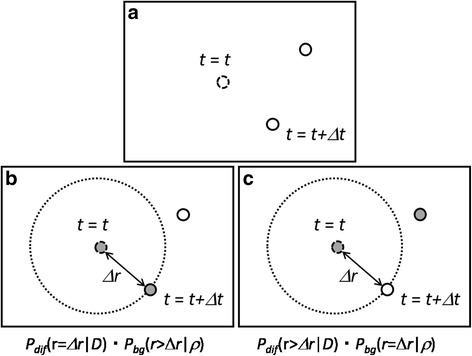
Schematic of the probabilistic model. **a** a typical distribution of particles at *t* + Δ*t* (thick circles) with an indication of the position of a representative particle at *t* (dashed circle). **b** the case where the nearest particle is the original particle. **c** the case where the nearest particle is a surrounding particle. Gray color indicates the identification of the original particle. The large dotted circles indicate the distance to the nearest particle. The distance to the nearest neighbor of the origin at the subsequent time frame is modeled by the probabilistic model with respect to the diffusion constant of the original particle and the particle density at the origin

To develop the probabilistic model for estimating the 2D lateral diffusion constants under high particle density, we focus on a single Brownian particle in a time frame (Fig. 1). Without loss of generality, we take the position of the particle as the origin of our polar coordinates. As is well known, the probability of finding the same Brownian particle at a position with a radial distance greater than Δ*r* after a time-lag Δ*t* is given by [22].

1 $$ {P}_{\mathrm{dif}}\left(r>\Delta r|D\right)={e}^{-\frac{\Delta {r}^2}{4D\Delta t}}, $$

where the parameter _*D*_ is the diffusion constant of the particle.

In typical time-lapse single molecular imaging of cells, particles are indistinguishable from one another. By assuming the independence of the dynamics of each particle, we can model the distribution of such indistinguishable surrounding particles by a local uniform density,*ρ*, which is a sort of mean field approximation of the surrounding particles. In this approximation, we can derive the probability of having the nearest surrounding particle at a distance greater than Δ*r* as follows. We begin with a finite case where there are, on average, *N* surrounding particles in the disk with a radius *R* around a point. We assume that the surrounding particles are uniformly distributed within the disk. If we consider a smaller disk with a radius Δ*r* inside the disk, the probability of a single surrounding particle being found outside of the smaller disk is 1 − *a*/*A*, where *a* = *π*Δ*r*^2^ and *A* = *πR*^2^ correspond to the areas of the smaller and bigger disks, respectively. Then, the probability that all the *N* surrounding particles are also found outside of the smaller disk is (1 − *a*/*A*)^*N*^. Assuming that *a* is much smaller than *A*, this probability can be approximated as

$$ {\left(1-\frac{a}{A}\right)}^N=\exp \left(N\log \left(1-\frac{a}{A}\right)\right)\cong \exp \left(-\frac{aN}{A}\right)=\exp \left(-\rho \pi \Delta {r}^2\right), $$

where *ρ* = *N*/*A* is the local particle density. Thus, the probability of having the nearest surrounding particle at a distance greater than Δ*r* is given by

2 $$ {P}_{\mathrm{bg}}\left(r>\Delta r|\rho \right)={e}^{-\rho \pi \Delta {r}^2}. $$

By combining the above results together, the probability of detecting the nearest particle at a distance greater than Δ*r* would be given by

3 $$ {P}_{\mathrm{nn}}\left(r>\Delta r|\rho, D\right)={P}_{\mathrm{dif}}\left(r>\Delta r|D\right){P}_{\mathrm{bg}}\left(r>\Delta r|\rho \right)={e}^{-\rho \pi \Delta {r}^2-\frac{\Delta {r}^2}{4D\Delta t}}. $$

This is the fundamental probabilistic model upon which we develop the estimation algorithm of the diffusion constant in this paper (Fig. 1). This probabilistic model generalizes the theory of Brownian motion of a single isolated particle into that of a single particle surrounded by indistinguishable particles.

The indication of the model becomes more manifest if we calculate the expected mean square displacement to the nearest particle (MSDN) as

4 $$ \mathrm{MSDN}=E\left(\Delta {r}^2\right)\equiv \underset{0}{\overset{\infty }{\int }}\Delta {r}^2\left(-\frac{d}{d\left(\Delta r\right)}{P}_{\mathrm{nn}}\left(r>\Delta r|\rho, D\right)\right)d\left(\Delta r\right)=\frac{4D\Delta t}{1+4\rho \pi D\Delta t}. $$

This is a natural generalization of the well-known relationship between the MSD of a single diffusing particle and the diffusion constant [22],

5 $$ \mathrm{MSD}=4D\Delta t. $$

As expected, MSDN goes back to the original MSD in the limit of *ρ* being zero, (i.e., where there are no surrounding particles). Due to the additional term in the denominator, the MSDN is, in general, smaller than MSD. This is because the nearest particle can be the original particle diffused from the origin as in MSD, or even a nearer surrounding particle.

This relationship can be easily solved with respect to *D*, allowing it to be estimated as

6 $$ D=\frac{\mathrm{MSDN}}{4\Delta t\left(1-\rho \pi \kern0em \mathrm{MSDN}\right)}. $$

**Fig. 2:**
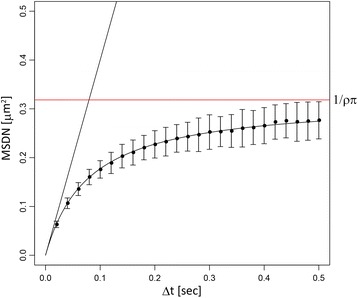
Mean square displacement to the nearest particle. A comparison of MSDN and MSD. The black straight line corresponds to the expected MSD, while the black curve is the expected MSDN, with *D*=1 μm^2^/s and *ρ*=1 particles/μm^2^. The points are the mean MSDN directly calculated from corresponding simulated data. The error bars indicate the standard deviation from one thousand independent simulations. The red line indicates the asymptotic value of the expected MSDN at Δ*t* → ∞

Compared to the standard estimation from MSD,

7 $$ D=\frac{\mathrm{MSD}}{4\Delta t}, $$

the estimated diffusion constant acquires a fold increase of 1/(1 − *ρπ*MSDN), which compensates for the apparent reduction of the displacement compared to MSD. In Fig. 2, we show the MSDN for simulated data. As Δ*t* increases, the points deviate from the line 4*D*Δ*t* and obey the above theoretical prediction as expected. Note that the time course of MSDN is conceptually different from that of MSD in a trajectory after SPT. In the case of SPT, the identification of the same particle is consecutively performed using all measured time points during Δ*t*. On the other hand, in MSDN, the nearest point after time duration Δ*t* was chosen without referring to the measured time points before Δ*t*.

### Maximum likelihood estimation of diffusion constants for local particle density

Though the above relationship between the diffusion constant and MSDN allows us to estimate diffusion constants for the case of a uniform particle distribution, it is difficult to generalize it into an inhomogeneous particle distribution, which is a less ideal but much more relevant situation. In such a case, a constant particle density *ρ* alone cannot capture the underlying particle distribution.

Here, we formulate a more general estimation algorithm of diffusion constants using a maximum likelihood estimation based on the above probabilistic model. The log-likelihood of an observed dataset is given by

8 $$ {\displaystyle \begin{array}{l}l=\log \prod \limits_{i=1}^N{P}_{\mathrm{nn}}\left(r=\Delta {r}_i|{\rho}_i,D\right)\\ {}=\sum \limits_{i=1}^N\left[\log \left(2{\rho}_i\pi +\frac{1}{2D\Delta t}\right)+\log \left(\Delta {r}_i\right)-{\rho}_i\pi \Delta {r}_i^2-\frac{\Delta {r}_i^2}{4D\Delta t}\right].\end{array}} $$

Here, the index *i* represents each particle in the preceding time frame, Δ*r*_*i*_ is the distance to the nearest particle in the subsequent time frame, and *ρ*_*i*_ is the local particle density around particle *i*. If we assume a uniform distribution (i.e., that all *ρ*_*i*_ are the same), this maximum likelihood estimation of *D* is analytically tractable and reduces to the same relation between the diffusion constant and MSDN described above.

In the case of general *ρ*_*i*_, it is convenient to utilize the EM algorithm [21, 23]. For this purpose, we introduce a latent variable *q*_*i*_ ∈ {0, 1}, which takes the value of zero if the nearest point comes from the surrounding particles, but becomes one if it is the original particle diffused from the origin. Then the complete-data log-likelihood with the information of the latent variable is given by

9 $$ {l}^{\prime }=\log \prod \limits_{i=1}^Np\left(\Delta {r}_i,{q}_i|{\rho}_i,D\right). $$

Here, the joint probability distribution is defined as

10 $$ p\left(\Delta {r}_i,{q}_i|{\rho}_i,D\right)=\left\{\begin{array}{cc}2{\rho}_i\pi \Delta {r}_i{e}^{-{\rho}_i\pi \Delta {r}_i^2-\frac{\Delta {r}_i^2}{4D\Delta t}}& \mathrm{for}\kern0.24em {q}_i=0\\ {}\frac{\Delta {r}_i}{2D\Delta t}{e}^{-{\rho}_i\pi \Delta {r}_i^2-\frac{\Delta {r}_i^2}{4D\Delta t}}& \mathrm{for}\kern0.24em {q}_i=1\end{array}\right.. $$

In the EM algorithm, instead of maximizing the log-likelihood directly, a quantity *Q*(*D*, *D*^*l*^) is maximized with respect to *D* by iteration:

11 $$ Q\left(D,{D}^l\right)=\sum \limits_{i=1}^N\sum \limits_{q\in \left\{0,1\right\}}\log \left(p\left(\Delta {r}_i,q|{\rho}_i,D\right)\right)p\left(q|\Delta {r}_i,{\rho}_i,{D}^l\right). $$

Here, *D*^*l*^ is the estimation of the diffusion constant *D* at the *l*-th iteration. The conditional probability based on *D*^*l*^ is calculated from the above joint probability as

12 $$ p\left(q=0|\Delta {r}_i,{\rho}_i,{D}^l\right)=\frac{4{\rho}_i\pi {D}^l\Delta t}{4{\rho}_i\pi {D}^l\Delta t+1}, $$

13 $$ p\left(q=1|\Delta {r}_i,{\rho}_i,{D}^l\right)=\frac{1}{4{\rho}_i\pi {D}^l\Delta t+1}. $$

Taking the derivative of *Q* with respect to *D* and equating it to zero,

14 $$ \frac{dQ}{dD}=\sum \limits_{i=1}^N\left[\frac{\Delta {r}_i^2}{4{D}^2\Delta t}p\left(q=0|\Delta {r}_i,{\rho}_i,{D}^l\right)+\left(\frac{\Delta {r}_i^2}{4{D}^2\Delta t}-\frac{1}{D}\right)p\Big(q=1|\Delta {r}_i,{\rho}_i,{D}^l\Big)\right]=0, $$

we obtain the update rule

15 $$ {D}^{l+1}=\frac{\left\langle \Delta {r}^2\right\rangle }{4\Delta t{\left\langle P\left(q=1\right)\right\rangle}_{D^l}}, $$

where we have defined the expected fraction of data points with *q* = 1 as

16 $$ {\left\langle P\left(q=1\right)\right\rangle}_{D^l}\equiv \frac{1}{N}\sum \limits_{i=1}^Np\left(q=1|\Delta {r}_i,{\rho}_i,{D}^l\right). $$

Now, the correction from the original MSD relation is neatly summarized by this expected fraction of the data whose nearest points come from the original particle diffused from the origin.

### Generalization to models with multiple diffusive states

In this subsection, we further generalize the maximum likelihood estimation of diffusion constants into the case where particles take multiple states with different diffusion constants. It has been revealed that some membrane proteins change their physical properties upon binding to other molecules or spontaneous change of their conformation, and that these changes can be inferred from the change of the diffusion constant in some cases [14, 15]. Here we consider this type of change of diffusion constants, which we shall refer as to the change of their states. In this paper, we only provide the solution for relatively simpler situations of the dynamics with multiple diffusive states where the interconversion of different states can be ignored in the time resolution [10].

This simple generalization is practically quite useful, even when there is no biological reason to expect the existence of such multiple states of the target molecule. In a real experiment, many fluorescently-dyed surface molecules disappear for several reasons, such as internalization of the particle, breaching of the fluorescent dye, and so on. Such disappearance of particles can be modeled in the above framework by adding an additional state whose diffusion constant is infinitely large. In addition, some accidental peaks of fluorescent intensity may be wrongly detected as particles due to the low signal-to-noise ratio of the original images (false detections). Those spurious particles also tend to disappear in the subsequent time frame. Thus, we can reduce the effects of such false detections by introducing such a state in advance. We will address this issue again in Result section.

The derivation of the corresponding EM algorithm is largely parallel to the one in the previous subsection. In addition to the latent variable *q*_*i*_, which specifies whether or not the nearest particles are the original particle itself, we introduce an additional latent variable specifying states of the particle *i*, *s*_*i*_ ∈ {1, ⋯, *M*}, where *M* is the number of possible states.

The joint probability distribution of this model is given by

17 $$ p\left(\Delta {r}_i,{q}_i,{s}_i|{\rho}_i,{D}_{s_i},{\alpha}_{s_i}\right)=\left\{\begin{array}{cc}2{\rho}_i{\pi \alpha}_{s_i}\Delta {r}_i{e}^{-{\rho}_i\pi \Delta {r}_i^2-\frac{\Delta {r}_i^2}{4{D}_{s_i}\Delta t}}& \mathrm{for}\kern0.24em {q}_i=0\\ {}\frac{\alpha_{s_i}\Delta {r}_i}{2{D}_{s_i}\Delta t}{e}^{-{\rho}_i\pi \Delta {r}_i^2-\frac{\Delta {r}_i^2}{4{D}_{s_i}\Delta t}}& \mathrm{for}\kern0.24em {q}_i=1\end{array}\right., $$

where ${D}_{S_i}$ is the diffusion constant of the state *s*_*i*_, and ${\alpha}_{s_i}$ is the probability of being the state *s*_*i*_.

The quantity *Q* for deriving the update rule of the EM algorithm is similarly defined by

18 $$ Q\left(D,{D}^l\right)=\sum \limits_{i=1}^N\sum \limits_{s=1}^M\sum \limits_{q\in \left\{0,1\right\}}\log \left(p\left(\Delta {r}_i,q,s|{\rho}_i,\theta \right)\right)p\left(q,s|\Delta {r}_i,{\rho}_i,{\theta}^l\right). $$

Here, *θ* collectively denotes all of the parameters to be estimated, namely, *θ* = {*D*_1_, ⋯, *D*_*M*_, *α*_1_, ⋯*α*_*M*_}. The conditional probability is calculated from the joint probability as follows:

19 $$ p\left(q=0,s|\Delta {r}_i,{\rho}_i,{\theta}^l\right)=\frac{2{\rho}_i{\pi \alpha}_s^l{e}^{-\frac{\Delta {r}_i^2}{4{D}_s\Delta t}}}{\sum \limits_{s^{\prime }=1}^M\left(2{\rho}_i\pi +\frac{1}{2{D_{s^{\prime}}}^l\Delta t}\right){\alpha}_{s^{\prime}}^l{e}^{-\frac{\Delta {r}_i^2}{4{D}_{s^{\prime }}\Delta t}}}, $$

20 $$ p\left(q=1,s|\Delta {r}_i,{\rho}_i,{\theta}^l\right)=\frac{\frac{\alpha_s^l}{2{D_{s^{\prime}}}^l\Delta t}{e}^{-\frac{\Delta {r}_i^2}{4{D}_s\Delta t}}}{\sum \limits_{s^{\prime }=1}^M\left(2{\rho}_i\pi +\frac{1}{2{D_{s^{\prime}}}^l\Delta t}\right){\alpha}_{s^{\prime}}^l{e}^{-\frac{\Delta {r}_i^2}{4{D}_{s^{\prime }}\Delta t}}}. $$

Compared to the single state case, here, the joint probability also depends upon the displacement, Δ*r*_*i*_.

By maximizing *Q* under the restriction of conservation of probability, $\sum \limits_s{\alpha}_s=1$, we obtain

21 $$ {\alpha_s}^{l+1}=\frac{1}{N}\sum \limits_{i=1}^N\sum \limits_{q\in \left\{0,1\right\}}p\left(q,s|\Delta {r}_i,{\rho}_i,{\theta}^l\right), $$

22 $$ {D_s}^{l+1}=\frac{\sum \limits_{i=1}^N\sum \limits_{q\in \left\{0,1\right\}}\Delta {r_i}^2p\left(q,s|\Delta {r}_i,{\rho}_i,{\theta}^l\right)}{4\Delta t\sum \limits_{i=1}^Np\left(q=1,s|\Delta {r}_i,{\rho}_i,{\theta}^l\right)}. $$

This is our final update rule for maximum likelihood estimation for the multi state model.

## Methods

### Monte Carlo simulation

To compare the performance of the proposed and existing methods, we generate artificial data of single molecular particle diffusion with Monte Carlo simulation. Depending on the purpose of simulation, we generate simulated data in two different ways.

### Pairwise simulation

In [16], to evaluate the performance of PICS algorithm, the authors utilized simulated data generated as pairs of time frames, rather than a single time course of diffusing particles. Since it allows precise controls of the distribution of the simulated data, it makes subsequent comparison among algorithms and interpretation of observed performance easier. Thus, we follow the same strategy to simulate diffusion dynamics in some of our simulations in Result section.

First we draw a fixed number of positions of particles from the corresponding probability distribution of particles for the preceding time frame. In the case of uniform particle distribution, we sample the particles over a much larger area than the area of interest, in order to keep the same distribution after the diffusion steps. Next, we generate the subsequent frame by adding a displacement drawn from the two-dimensional normal distribution with a variance of 2*D*Δ*t* to each position. When needed, another fixed number of particles are drawn from the same particle distribution, and added independently to both the preceding and subsequent frames to represent the existence of false detections, which typically occur in detection from low signal-to-noise ratio image data. In the simulation with false detections, we set the fraction of false detections to 20%. Each estimation of diffusion constants is performed against 10 pairs of time frames. The simulation is repeated 100 times for each condition. All simulations are performed using R (http://www.r-project.org/).

### Image based time course simulation

In the above simulation method, positions of detected points were directly generated by Monte Carlo simulation. Thus, no particular bias coming from detecting particle positions from image data is taken into account. In order to take account of such uncontrollable effects, we further examine diffusion constant estimation algorithms by artificially generated time course image data of single molecular measurements. For this purpose, we utilize the image data generator provided as a plugin “ISBI Challenge Track Generator” [24] of an open platform software “ICY” [25] for bioimage analysis.

We set the parameters of the plugin software as follows; SNR = 4, sequence length = 10, particle density = 100, 500 and 1000, sigma = 1, 2, 3, 5, 7 and 10 in the particle motion with creator type “BROWNIAN_UNIFORM”. The image size is 512 pixels × 512 pixels. The other parameters (except for seeds) are set to default, which means the extinction rate of each particle is 0.05. The particles in generated image data are detected by another plugin “Spot Detector” of the ICY software. The detection of bright spots by Spot Detector plugin is performed with default parameters. The simulation is repeated 3 times for each condition with different seed values.

### Other algorithms to estimate diffusion constants

To evaluate the performance of our proposed method, we compare it with existing algorithms. To make the comparison make sense, we examine algorithms that are applicable to the same type of the data, namely, the time series of the location of detected points. For example, some of algorithms utilized to estimate the diffusion constants under higher density or higher diffusion speed cannot be compared because they require specially designed data set for the algorithms [26, 27]. As a result, our comparison is made mainly with PICS algorithm which is particularly designed for estimating diffusion constants under higher particle density, in addition to SPT based methods.

#### PICS

We implement the PICS algorithm in R to enable automatic parameter estimation from the Monte Carlo simulation data. A minor difference from the original implementation described in [16] is that we fit the whole cumulative correlation function at once to simplify the automation instead of separately fitting the linear and non-linear parts of the cumulative correlation function to the data. In our experience, this implementation of PICS provides comparative or even better performance compared to the original one (data not shown).

#### Local SPT

As an example of the most naïve approach, we make trajectories by simply associating each particle to the nearest particle in the subsequent frame without considering global consistency. Unlike the case of global SPT described below, in this approach, a particle in a subsequent time frame might be associated with several particles in the preceding time frame.

#### Global SPT

As a representative of SPT method, we implement the global linking algorithm based on a greedy hill-climbing optimization with topological constraints following the literature [24, 28]. This algorithm was used in one of the best performance groups in the international competition of particle tracking methods [24]. In this algorithm, there is no conflict between the associations of each particle. We set the maximum distance parameter for limiting the association of subsequent particles to a large enough value to link all particles. For the pairwise simulation, this procedure provided sensible estimation of diffusion constants independent of the details of the exact value of the maximum distance parameter, as far as the particle density is not very high (data not shown).

After obtaining the distribution of diffusion step sizes with local or global SPT, we estimate the diffusion constant with a maximal likelihood estimation based on the assumption that each single particle exhibits Brownian motion.

### Particle density estimation for the simulated data

To apply our algorithm, we have to estimate the (local) particle density. In the case of a uniform distribution, we estimate the density by simply dividing the total particle number in the frame by the area of interest. In an inhomogeneous case, it is difficult to accurately estimate the local particle density based on just a single time frame. Therefore, we estimate the local probabilistic density by a k nearest-neighbor algorithm after merging all subsequent frames in the dataset except for the one in the frame of interest. Then, the particle density at the point is obtained by weighting the probabilistic density with the number of particles in the frame of interest. The value of *k* from the k nearest neighbor density estimation in the merged data is chosen to be the number of time frames utilized, which corresponds to the length scale of *k* = 1 in a time frame.

### Estimation of diffusion constants for the real data

HeLa cells grown on glass coverslips (Matsunami) in a 6-well plate were transfected with Lyn_11_-Halotag using Lipofectamine 2000 (Invitrogen). Afters 4 h, the culture medium was replaced with DMEM and the cells were incubated at 37 °C for 24 h. The culture medium was exchanged with Opti-MEM (Gibco), and the cells were incubated at 37 °C. After 2 h, the cells were washed once with OPTI-MEM and incubated with 0.03 nM of Halotag TMR ligand (Promega) in Opti-MEM for 30 min in a CO2 incubator. The cells were then washed three times with Opti-MEM and single-molecule imaging was performed using a TIRF microscope. Single particle detection and estimation of diffusion constants were done using ICY and PNN algorithm, respectively.

## Results and Discussion

### Dependence of estimated diffusion constants on particle density

Both PICS and our estimation algorithm, hereafter called the probabilistic nearest neighbor (PNN) estimation, have been designed to accurately estimate diffusion constants under the condition of high particle density. We first compare these methods to SPT-based methods with and without global optimization of linking (referred to as global SPT and local SPT, respectively) with pairwise simulated data (see Method section for details).

**Fig. 3:**
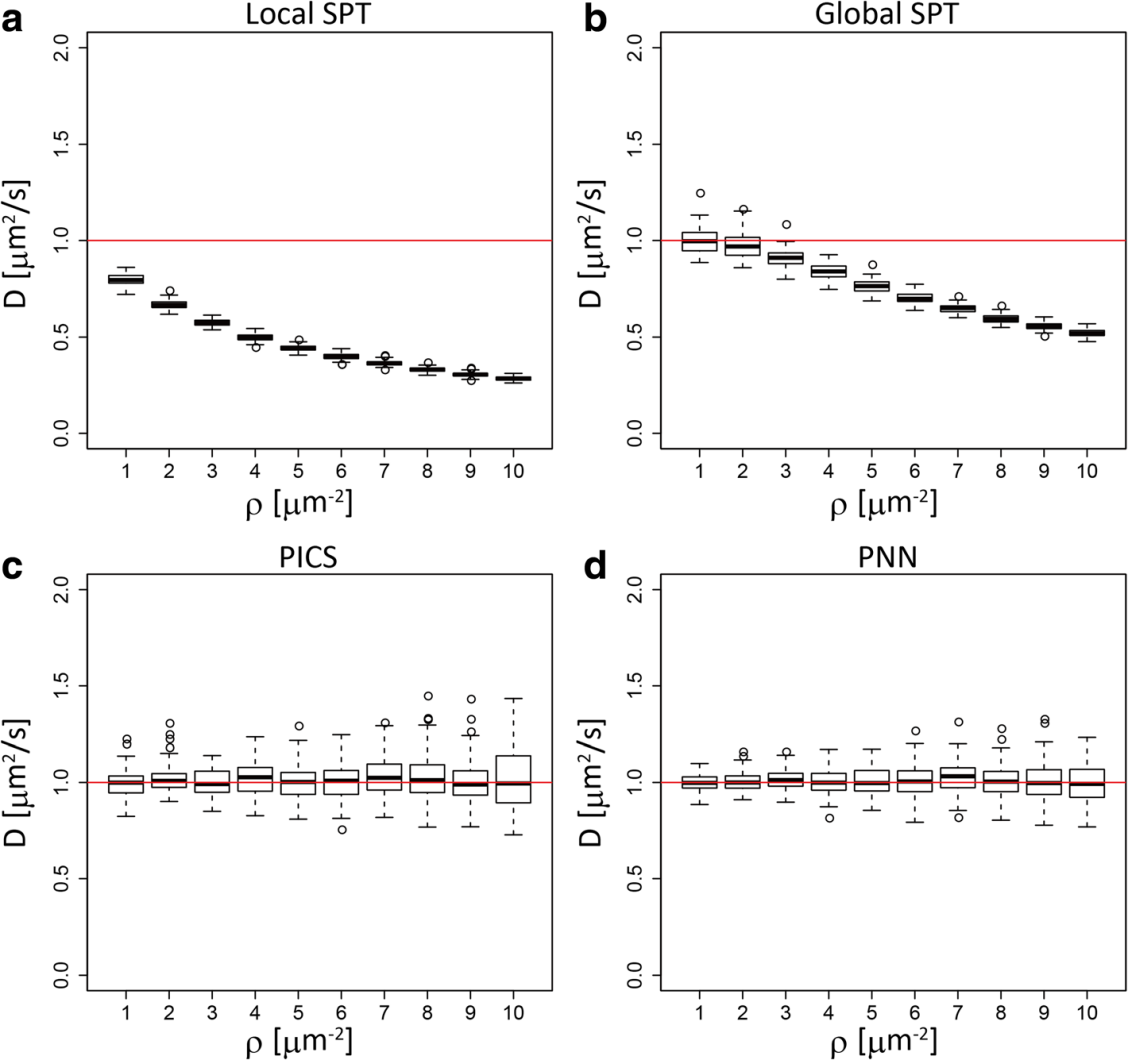
Comparison of the performance of different algorithms in a uniform distribution. Box plots summarizing a comparison of the algorithms. The x axis is the particle density and the y axis is the estimated diffusion constant. The red line indicates the true diffusion constant. **a** local SPT. **b** global SPT. **c** PICS and **d** PNN

First, we examine the effect of particle density under the ideal condition of a homogeneous distribution (Additional file 1: Figure S1 and Fig. 3). We vary the particle density from 0.1 to 10 particles/μm^2^, fixing the diffusion constant to be 1μm^2^/s. The time resolution, Δ*t*, of the data acquisition is assumed to be 20 ms [16]. Note that, in this ideal situation of Brownian motion, only the ratio of the scales of the diffusion constant and the particle density is the relevant parameter. Thus, the effects of changing the particle density with a fixed diffusion constant are effectively equivalent to the ones of changing the diffusion constant with a fixing particle density.

As expected, the change in particle density significantly affects the diffusion constants estimated by the simplest method, local SPT (Fig. 3). In this method, each pair of nearest neighbor points in the subsequent time frame is simply identified as the same physical particle without consideration of the behaviors of other particles. With this simple method, even with one-order lower particle density, the estimation accuracy is low due to the bias caused by the linking error (Additional file 1: Figure S1).

After global optimization (global SPT) of the linking, the estimation accuracy of SPT method is improved. In particular, under lower particle density conditions, it reproduces the true diffusion constants to great accuracy (Additional file 1: Figure S1). However, in the condition with higher particle density (*ρ* ≥ 2), this method also underestimates the diffusion constants. This value of the particle density roughly corresponds to that where 4*ρπD*Δ*t* becomes comparable to 1 in Eq. 1. This result suggests the limitation in SPT methods under high particle density conditions.

On the other hand, the two SPT-free methods PICS and PNN, which take the effects of surrounding particles explicitly into account, estimate the diffusion constants quite well over the whole range of particle densities under consideration (Fig. 3 and Additional file 1: Figure S1). Though the standard deviations among independent simulations tend to increase along with the increase of particle density, these could be reduced if more data in the same condition became available [16].

Thus, the estimation of diffusion constants using PICS or PNN leads to similar performance with SPT-based methods under lower particle density and outperforms them under higher particle density. Therefore, we focus on these two methods in the following discussion.

### Effect of false detections

By comparing PNN and PICS from the above results, one might conclude that the accuracy of PNN is slightly better than that of PICS because the standard deviation of the estimated results is smaller in the former than the latter. However, the above comparison was performed based on simulation in a quite ideal condition: particles distributed uniformly without any false detection. On the other hand, real single molecular measurements tend to be performed under less ideal conditions with a lower signal-to-noise ratio. This affects the accuracy of the detection of peak positions from raw images, leading to spurious particles that are wrongly detected in such noisy images.

**Fig. 4:**
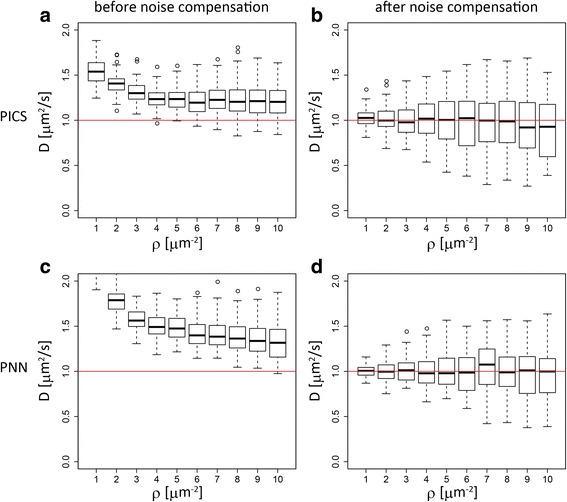
Comparison of the performance of PICS and PNN in a uniform distribution with false detections. Box plots summarizing the comparison of PICS (**a** and **b**) and PNN (**c** and **d**). The top row is for PICS and the bottom row is for PNN. The first column is the result before introducing the state corresponding to the false detections. The second column is the result after introducing the state for false detection compensation. The x axis is the particle density and the y axis is the estimated diffusion constant. The red line indicates the true diffusion constant

In order to mimic such a situation, we artificially introduce additional particles independently drawn from the same distribution in each time frame. We simply refer to these additional particles as false detections. The existence of false detections significantly degrades the estimation accuracy (Fig. 4, left panels) of both PNN and PICS. The effects of false detections in the diffusion constant estimation are two-fold. One effect is to increase the apparent density of surrounding particles in the subsequent time frames, and the other is the addition of spurious particles in the preceding time frames that immediately disappear from the scope. The former effect is, by design, treated both in PICS and PNN since the particle density is estimated with both physical particles and false detections. On the other hand, the spurious particles coming from false detections in the preceding time frames behave like particles with an infinitely high diffusion constant. Therefore, the addition of false detections biases the estimated diffusion constants towards higher values. Note also that similar effects may occur when actual particles disappear by internalization or dissociation of surface protein from the membrane, bleaching of fluorescent dye and so on.

Fortunately, as commented in Theory section, this effect of false detections can be addressed by generalizing the probabilistic model both in PICS and PNN by introducing an additional state for false detections with an infinitely large diffusion constant. With this generalization, both PICS and PNN improve their prediction accuracy (Fig. 4, right panels) with a cost of larger standard deviation, which originates from the increase of the number of the parameters to be estimated, namely the fraction of false detections.

### Estimation with an inhomogeneous distribution

As mentioned above, another idealization in the above simulation was the assumption of a uniform distribution of the particles. In fact, this is one of the key assumptions in the PICS algorithm. On the other hand, we have designed PNN to be applicable beyond this assumption. Here, we compare the performance of these two methods under three inhomogeneous distributions: Gaussian, circular and Gaussian mixture.

**Fig. 5:**
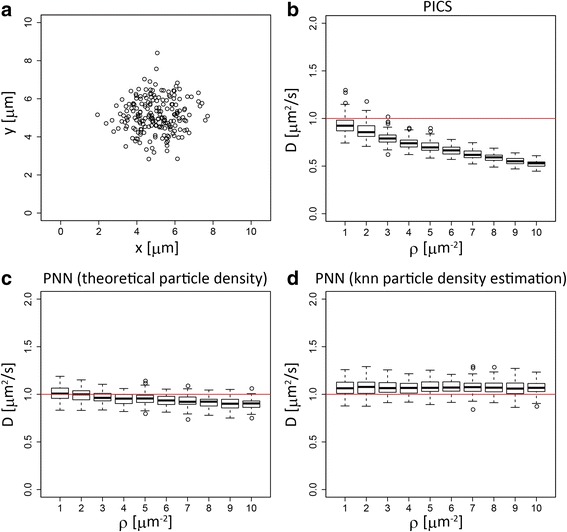
Comparison of the performance of PICS and PNN in a Gaussian distribution. **a** a representative snapshot of the particle distribution. **b**, **c**, and **d** box plots summarizing the comparison between PICS and PNN under a Gaussian distribution. **b** PICS. **c** PNN, where the known particle density distribution for the simulation is used for the diffusion constant estimation. **d** PNN where the particle density distribution is estimated from the data using a k nearest neighbor algorithm. The x axis is the mean particle density over the area of interest, and the y axis is the estimated diffusion constant. The red line indicates the true diffusion constant

Figure 5, Additional file 2: Figure S2 and Additional file 3: Figure S3 show the results of estimation of diffusion constants under three classes of inhomogeneous distributions, a Gaussian distribution, a circular distribution forming an annulus and Gaussian mixture distributions, respectively. Panel B of each figure shows the results of PICS, where the estimated diffusion constants are biased, especially for the higher particle density. This result is more or less expected, since this type of inhomogeneous condition is beyond the original scope of PICS.

Panel C of each figure is the result of the PNN estimation with the known theoretical distribution utilized to generate simulated data. In this case, the estimated diffusion constants are much closer to their true values. Of course, in a real situation, we cannot access to the true underlying distribution of the particles. Thus, we have to estimate the distribution from the data, and the accuracy of the diffusion constant estimation depends upon the accuracy of the density estimation. However, the results here demonstrate that as far as the particle density is estimated accurately enough, PNN should work reasonably well.

Panels D of Fig. 5, Additional file 2: Figure S2 and Additional file 3: Figure S3 show the results of PNN with a particle density estimated from the data itself. Here, in order to estimate the particle density, we use k nearest neighbor estimation. In general, there is a tradeoff between spatial resolution and statistical error in density estimation. Since our algorithm of PNN relies on the (first) nearest neighbor, smaller *k* values with high spatial resolution would be preferable. However, density estimation based on a smaller *k* tends to have a larger variance. In order to circumvent this problem, we estimate the particle density using all the post frames in the dataset except for the one in the frame of interest while keeping the effective *k* value equal to one (see Method section for details). The accuracy of the resultant diffusion constant is comparable to the accuracy using theoretical distributions. Our result here demonstrates that, with a suitable choice of density estimation methods, our algorithm can be utilized to estimate the diffusion constant, even under an inhomogeneous particle distribution.

### Image based simulation

To mimic a realistic situation of diffusion constant estimation from typical single molecular measurements, we further examine our algorithm and others using artificial image data generator for an open competition of SPT organized in 2012 [24]. The image data generator is provided as a plugin “ISBI Challenge Track Generator” of an open platform “ICY” for bioimage analysis. We generate image data of diffusion dynamics as triplicates for each condition. We set the parameters of the simulator to be relatively low signal-to-noise ratio, and short sequence length, to increase the difficulty of the estimation in the category of “BROWNIAN_UNIFORM” (see Method section for details). Note also that the particles in this simulation disappear with an extinction rate of 0.05. A representative movie and images of this simulation are in Additional file 4: Movie S1 and in Additional file 5: Figure S4, respectively. The detection of the particles from image data was made by another plugin “Spot Detector” of the ICY software.

**Fig. 6:**
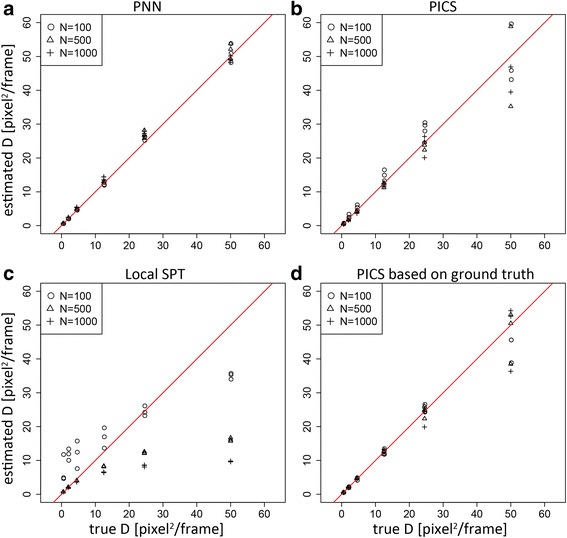
The performance of different algorithms in image based simulations. Scatter plots summarizing the performance of the algorithms. The x axis is the true diffusion constant used for the simulation and the y axis is the estimated diffusion constant. The red line indicates the diagonal line corresponding to the successful estimation. **a** PNN. **b** PICS. **c** local SPT and **d** PICS applied to the corresponding ground truth data

The results of the estimation of diffusion constants are summarized in Fig. 6. Here, we show the diffusion constants estimated by PNN, PICS and Local SPT. Though we have also applied global SPT to the same data, it showed very strong dependence on the maximum distance parameter and we could not obtain sensible estimation from the analysis (data not shown). Thus, the results of global SPT are omitted. We observe very similar tendency as in the previous simulations. PNN provides the most accurate results over the range of simulated conditions. Local SPT shows very strong bias depending on the particle density (number) and true diffusion constants. PICS does not show particular bias but tends to have higher variances. Visual inspection of the fitted curves of PICS clearly indicated poor fitting due to the effect of diffraction, as discussed in the original paper of PICS [16]. In the paper, they discussed how to mitigate the effect of diffraction in an iterative algorithm. Here, instead of implementing their iterative algorithm, we apply PICS to the corresponding ground truth data provided by the simulator, which are free from all the effects of diffraction (Panel D). Though this additional favor improves the fitting and the performance of PICS, PNN still seems to outperform the ground truth based PICS (Fig. 6). These results indicate the advantage of PNN in the application to the real image data from single molecular measurement of living cells.

### 3D visualization of particle states

**Fig. 7:**
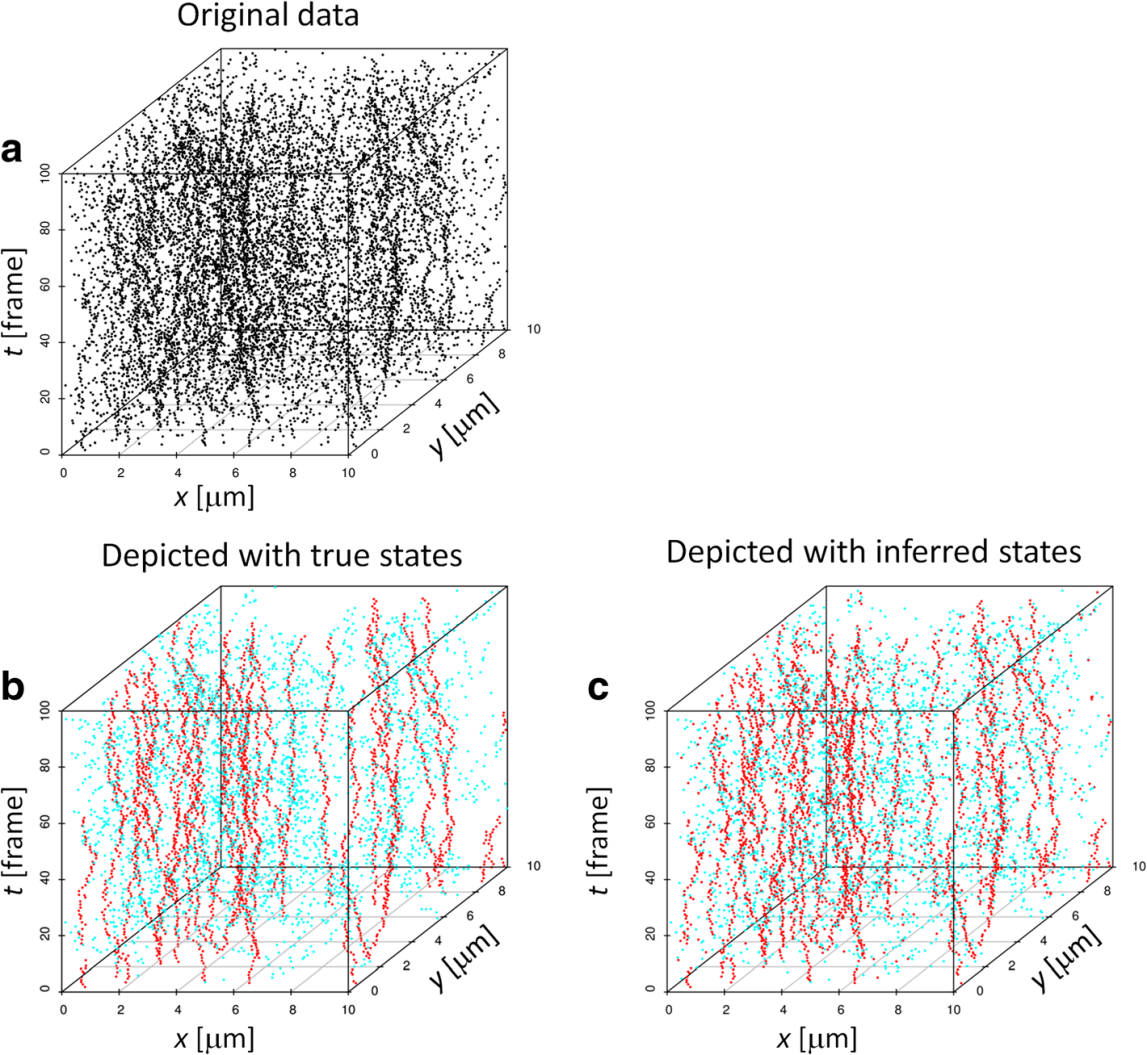
3D visualization of particle positions and states. 3D representation of the time course simulated data of diffusing particles. The z axis corresponds to time while the other two axes correspond to the x- and y-axes of the original data. **a** the original data. **b**, the same data depicted in color (red: slower particle (0.2 μm^2^/s), cyan: faster particle (2 μm^2^/s)) after removing the false detection. **c** the same data depicted in colors based on the particle states inferred by PNN

The key of the proposed algorithm is that it assigns a probability of taking each possible state to each particle detected without specifying a trajectory. This property of the algorithm can be utilized to visualize time course data itself. The data shown in the upper panel of Fig. 7 consists of particles taking three different states, namely slower diffusion (0.2 μm^2^/s), faster diffusion (2 μm^2^/s), and false detections. The particle density including all of the three states is 1 particles/μm^2^. The lower left panel is the same data in color (red: slower particle, cyan: faster particle) after removing the false detections. We apply the PNN algorithm to the data and infer the state of each particle by choosing the most probable one among the assigned probabilities. As shown in the lower right panel, the resultant figure bears a strong resemblance to the original data, giving another support for the validity of this algorithm. Unlike canonical SPT methods attempting to determine a hard-wired trajectory, our algorithm keeps several possibilities at the same time. This application of PNN to a visualization purpose would be useful, particularly when one is interested in identifying rare events like interactions between pairs of particles.

### Application to real data

We applied the PNN algorithm to a real data. Lyn_11_-Halotag construct, which is localized on cytoplasmic membrane, was expressed in HeLa cells and single-molecule imaging was performed (Additional file 6: Movie S2). Particle detection data was generated by using ICY and subjected to estimation of diffusion constant by PNN. PNN with *k* nearest neighbor particle density estimation resulted in diffusion constant of 2.81 × 10^− 2^ μm^2^/s under the assumption where molecules take only one state and those of 5.20 × 10^− 3^ μm^2^/s and 6.15 × 10^− 2^ μm^2^/s under the assumption where molecules take two states. We could also calculate AIC under these assumptions and the result supported the latter. In the case, fractions of false detection, slow and fast states were estimated to be 36%, 24% and 40%, respectively. A previous report [29] suggested that Lyn, origin of the Lyn_11_ tag, exhibits two states, namely lateral diffusion and transient confinement in a lipid region through lipid-lipid interaction. This supports our result where Lyn_11_-Halotag has two states with slow and fast diffusion. Together, this result suggests that the proposed algorithm works well for real data and helps to understand dynamics of molecules.


Additional file 6:**Movie S2.** TIRF microscopic single molecule video image of Lyn_11_-Halotag in HeLa cells. Membrane-localized single Lyn_11_-Halotag protein molecules in a HeLa cell were observed by a TIRF microscope as described in Methods. (AVI 2796 kb)


## Conclusions

In this paper, we proposed a novel diffusion constant estimation algorithm based on a probabilistic model of the nearest point without explicitly performing SPT. Though conventional SPT methods try to link pairs of particles in the subsequent frames in a hard manner, such hard linking inevitably leads to erroneous pairing if no other information to distinguish particles is available. We have derived a probabilistic model by explicitly considering a Brownian particle surrounded by indistinguishable particles in a mean field approximation. Since our probabilistic model allows us to estimate diffusion constants without relying on particular hard-linked trajectories, it performs well even in the cases with higher particle density or higher diffusion speed, where standard SPT methods underestimate the diffusion constant. Since particle density is difficult to control in real experiments, this is advantageous in practical usage.

We have also provided a generalization of our algorithm to multiple diffusive states. This generalization was the key to address the case with false detections, since disappearing particles behave like particles with the additional diffusive state whose diffusion constant is infinity. Thus, in practice, one is recommended to examine both models with and without a fraction of disappearing particles, and select a model by comparing a statistical indicator like the Akaike Information Criterion [30].

In addition to high prediction accuracy, one of the advantages of PNN is its applicability beyond a uniform particle distribution. This has been the limitation on PICS, another existing SPT-free algorithm. We have demonstrated that, with or without knowledge of the underlying distribution, our algorithm accurately estimates diffusion constants even for the cases where PICS cannot be properly applied. In general, without prior knowledge of the underlying particle distribution, the actual performance of diffusion constant estimation also depends upon the accuracy of the estimation of the underlying particle distribution from the data, though the investigation of optimal density estimation itself is beyond the scope of this paper.

Since PNN considers each particle separately, it allows us to obtain detailed information about each particle. With the help of the EM algorithm, PNN estimates the probability that each particle is in each state. This kind of information, combined with their spatial distribution, can be used for providing further insights into the underlying biology, as briefly demonstrated in Fig. 7.

Another advantage of the proposed method, which is not apparent from the above benchmark results, is the small number of hyperparameters to be determined before analyses. For example, SPT based methods typically have a hyperparameter corresponding to the maximum distance parameter, which specifies the possible maximum displacement of diffusing particles to avoid connections of completely irrelevant particles. As mentioned in Result section, the estimated diffusion constants tend to largely depend on the choice of such a hyperparameter especially when particle density or diffusion speed is higher. PICS also requires several number of hyperparameters to perform a fitting to the empirical cumulative correlation functions [16], including the bin size and the range of consideration, whose optimal values may depend on data. On the other hand, PNN under a homogeneous particle distribution effectively has only a single hyperparameter, the margin to define the range of interest of the preceding time frames compared to the subsequent time frames, which also needed for PICS in addition to the ones mentioned above. We have confirmed that PNN has very weak dependency on the margin parameter as expected from the construction of the algorithm (data not shown). In the case of PNN under inhomogeneous particle distribution, the number of hyperparameters may vary depending on the chosen method of particle distribution estimation. In fact, in combination with the k nearest neighbor estimation of particle distribution we utilized in this paper, no hyperparameter, even the margin parameter, is required. This nature of small number of hyperparameters in PNN is very convenient in practice, since, otherwise, many trials and errors are needed to optimize hyperparameters. In particular, when the absolute value of estimated parameters is of concern, it is not a trivial matter to choose such hyperparameters objectively.

Finally, we would like to emphasize the complementary role of diffusion constant estimation methods. First of all, all of the methods we examined in this paper are based on the assumption that identification of single molecules from the image data is more or less possible. If the particle density is too high, the resultant images cannot have the resolution of single molecular imaging. In this extreme case, other methods without relying on particle detection at all, like image correlation microscopy [17–20], would be preferable. Or, if one can use specially designed experimental equipments, some other choices of methods to finely estimate diffusion constants like [26, 27] are available. In the case of the typical time lapse images of single molecules [3, 4, 12–15] which we considered in this paper, we have demonstrated that PNN is favorable than PICS, in terms of the accuracy, the applicability to inhomogeneous distribution and the convenience of the analysis due to the small number of hyperparameters. However, PICS analysis also has an advantage that the analysis is more graphical than PNN and one might be able to address the validity of the model by a visual inspection as far as the underlying spatial distribution of the particle is uniform. In turn, though canonical SPT methods tend to underestimate the diffusion constant and largely depend on hyperparameters under higher particle distribution, they allow one to analyze individual trajectories which may provide otherwise inaccessible information of each trajectory. In this sense, these methods can be utilized in combination. For example, one may first apply PNN to robustly estimate diffusion constants. This information of diffusion constants in turn might be utilized to determine the hyperparameters of SPT methods to minimize the linking error of SPT. Then, the resultant trajectories may be utilized, not to estimate the diffusion constants any more, but to extract other biologically interesting parameters which PNN cannot infer. Thus, having different diffusion estimation algorithms enlarges our freedom to analyze data, and increases the chance of obtaining biologically meaningful information from various single molecular time course datasets. In this regard, our algorithm opens a new window for accessing diffusion constants, in particular, in the regime where the particle density becomes comparable to the effective scale of diffusion.

## Additional files

Additional file 1: **Figure S1.** Comparison of the performance of different algorithms in uniform distributions with lower particle densities. Box plots summarizing the comparison of the algorithms as in Fig. 3. The x axis is the particle density and the y axis is the estimated diffusion constant. The red line indicates the true diffusion constant. A, local SPT. B, global SPT. C, PICS and D, PNN. (PNG 105 kb)

Additional file 2: **Figure S2.** Comparison of the performance of PICS and PNN in a circular distribution. A, a representative snapshot of the particle distribution. B, C, and D, box plots summarizing the comparison between PICS and PNN under a circular distribution. B, PICS. C, PNN, where the known particle density distribution for the simulation is used for the diffusion constant estimation. D, PNN where the particle density distribution is estimated from the data using the k nearest neighbor algorithm. The x axis is the mean particle density over the area of interest, and the y axis is the estimated diffusion constant. The red line indicates the true diffusion constant. (PNG 171 kb)

Additional file 3: **Figure S3.** Comparison of the performance of PICS and PNN in Gaussian mixture distributions. A, a representative snapshot of the particle distribution. The red crosses represent centers of three Gaussian distributions. B, C, and D, box plots summarizing the comparison between PICS and PNN under a Gaussian mixture distribution. B, PICS. C, PNN, where the known particle density distribution for the simulation is used for the diffusion constant estimation. D, PNN where the particle density distribution is estimated from the data using the k nearest neighbor algorithm. The x axis is the mean particle density over the area of interest, and the y axis is the estimated diffusion constant. The red line indicates the true diffusion constant. (PNG 306 kb)

Additional file 4: **Movie S1.** A representative movie of the image based simulation. A representative movie generated by the plugin, ISBI Challenge Track Generator, of an open platform software ICY. Seed = 123,456, SNR = 4, sequence length = 10, particle density = 1000, sigma = 10 in the particle motion with creator type “BROWNIAN_UNIFORM”. The other parameters are set to default, which means the extinction rate of each particle is 0.05. (TIFF 2561 kb)

Additional file 5: **Figure S4.** Representative images of the image based simulation. Representative images generated by the plugin, ISBI Challenge Track Generator, of an open platform software ICY. Seed = 123,456, SNR = 4, sequence length = 10, sigma = 10 in the particle motion with creator type “BROWNIAN_UNIFORM”. Particle densities are 100, 500 and 1000, respectively. The other parameters are set to default. (PNG 517 kb)
