# Supplementary figures and images for: Estimation of diffusion constants from single molecular measurement without explicit tracking
Source: BMC Syst Biol. 2018 Apr 11;12(Suppl 1):15. doi: 10.1186/s12918-018-0526-5 (PMC5907143; doi:10.1186/s12918-018-0526-5)

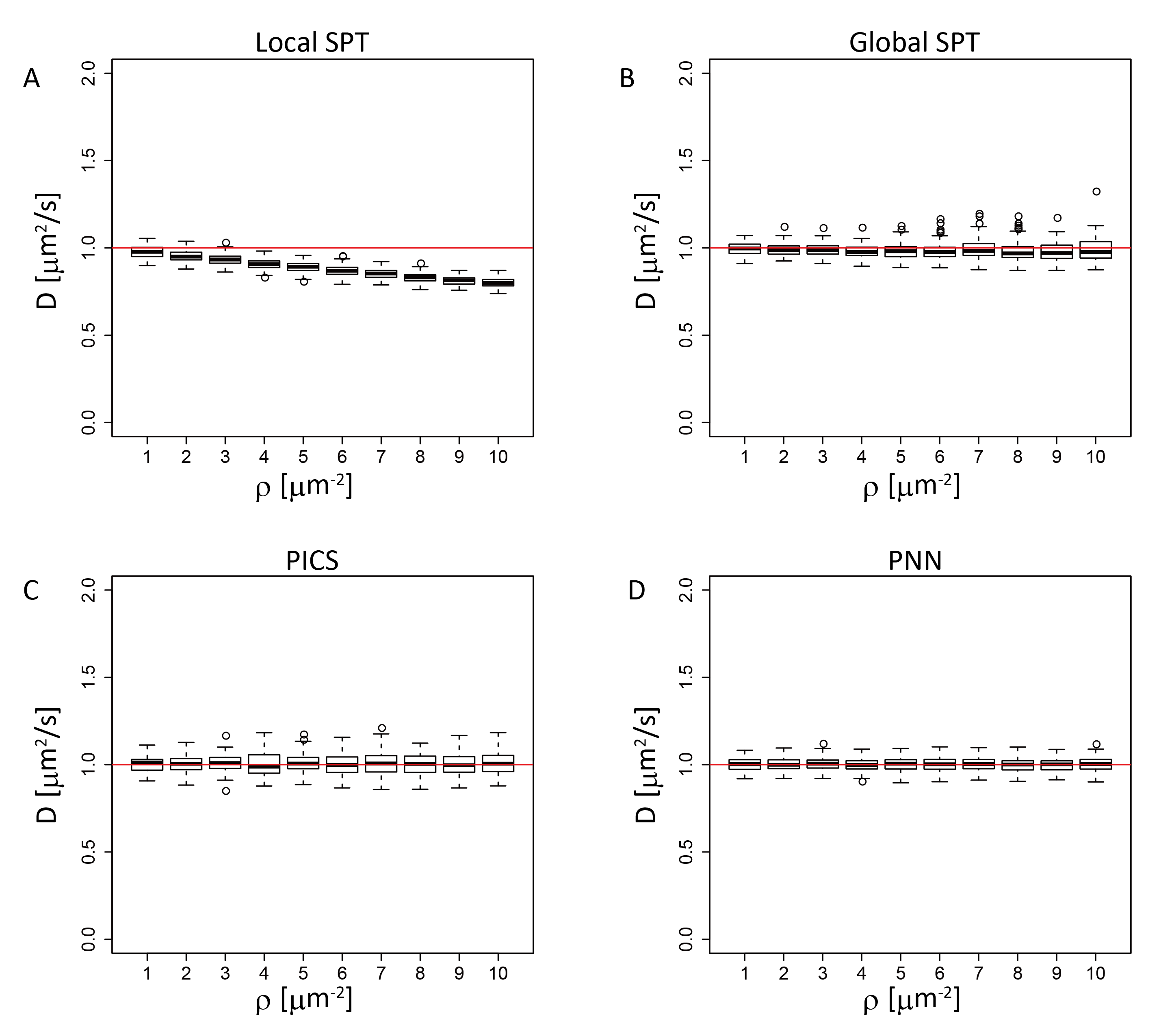

Supplement: Supplementary file 2 — Figure S1. Comparison of the performance of different algorithms in uniform distributions with lower particle densities. Box plots summarizing the comparison of the algorithms as in Fig. 3. The x axis is the particle density and the y axis is the estimated diffusion constant. The red line indicates the true diffusion constant. A, local SPT. B, global SPT. C, PICS and D, PNN. (PNG 105 kb) [file 12918_2018_526_MOESM1_ESM.png]

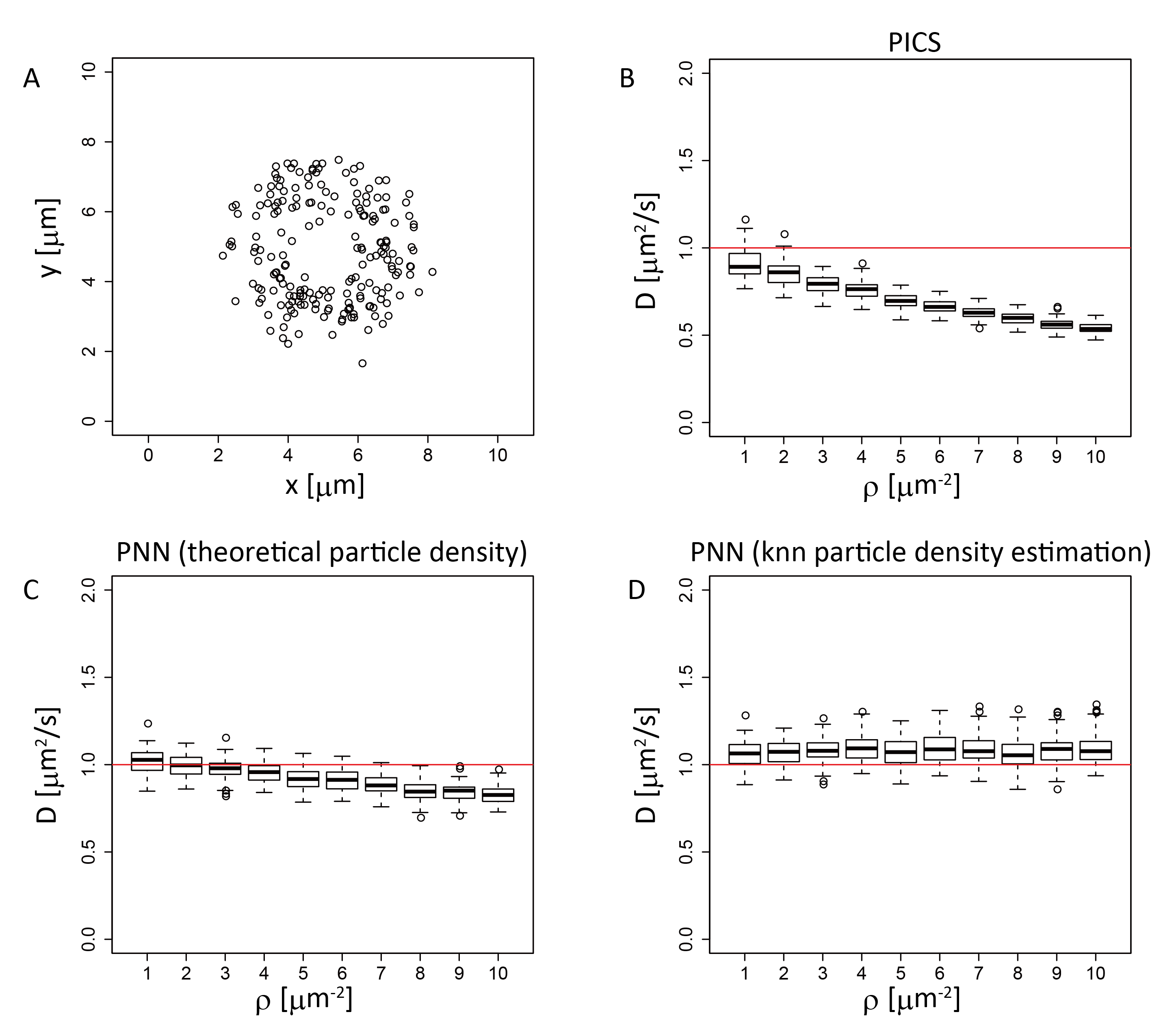

Supplement: Supplementary file 3 — Figure S2. Comparison of the performance of PICS and PNN in a circular distribution. A, a representative snapshot of the particle distribution. B, C, and D, box plots summarizing the comparison between PICS and PNN under a circular distribution. B, PICS. C, PNN, where the known particle density distribution for the simulation is used for the diffusion constant estimation. D, PNN where the particle density distribution is estimated from the data using the k nearest neighbor algorithm. The x axis is the mean particle density over the area of interest, and the y axis is the estimated diffusion constant. The red line indicates the true diffusion constant. (PNG 171 kb) [file 12918_2018_526_MOESM2_ESM.png]

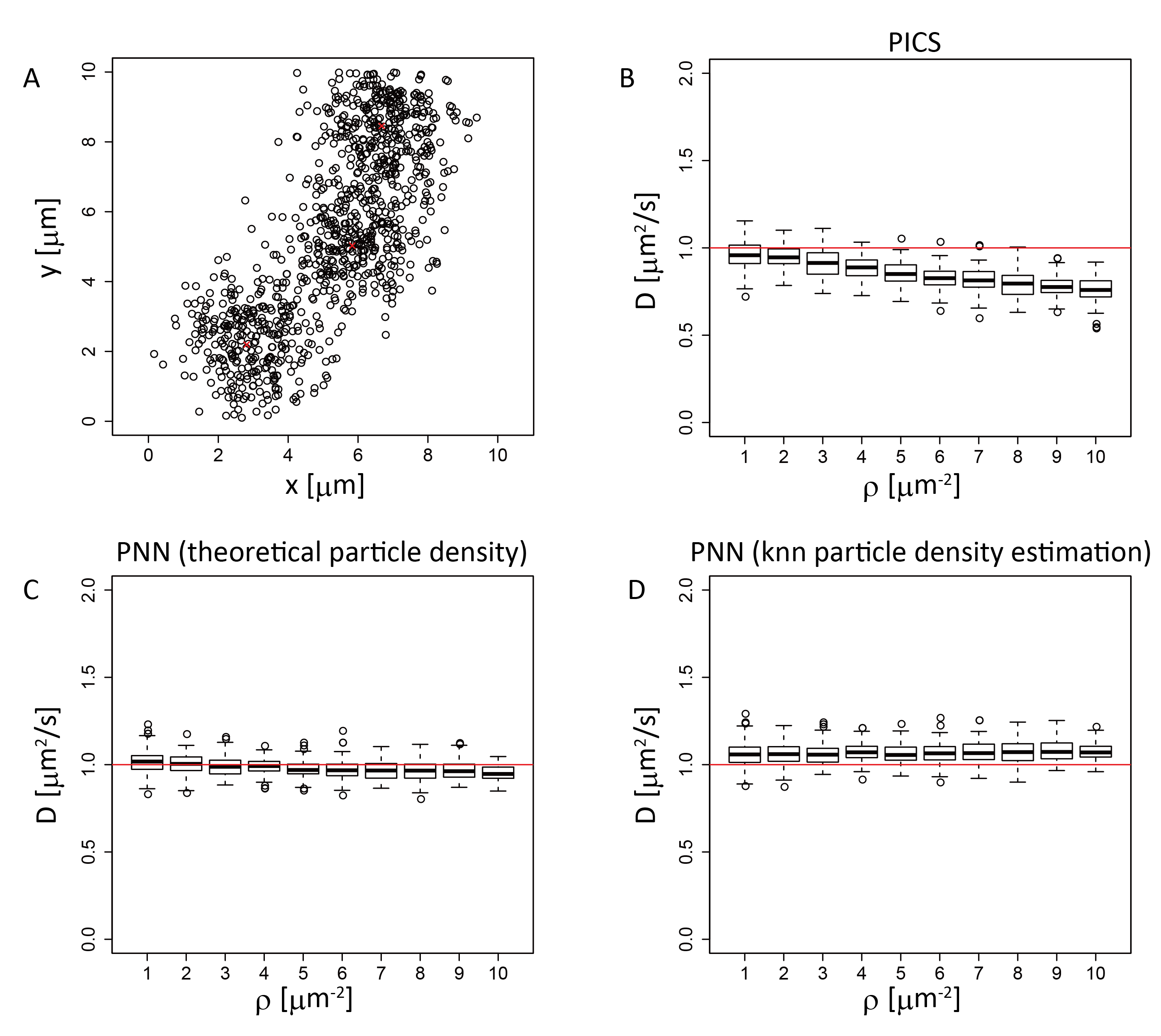

Supplement: Supplementary file 4 — Figure S3. Comparison of the performance of PICS and PNN in Gaussian mixture distributions. A, a representative snapshot of the particle distribution. The red crosses represent centers of three Gaussian distributions. B, C, and D, box plots summarizing the comparison between PICS and PNN under a Gaussian mixture distribution. B, PICS. C, PNN, where the known particle density distribution for the simulation is used for the diffusion constant estimation. D, PNN where the particle density distribution is estimated from the data using the k nearest neighbor algorithm. The x axis is the mean particle density over the area of interest, and the y axis is the estimated diffusion constant. The red line indicates the true diffusion constant. (PNG 306 kb) [file 12918_2018_526_MOESM3_ESM.png]

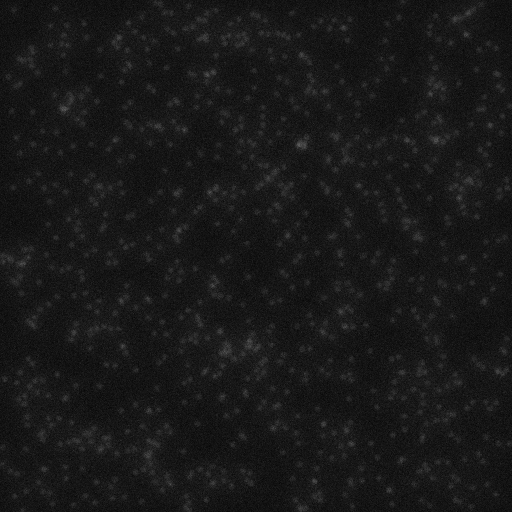

Supplement: Supplementary file 5 — Movie S1. A representative movie of the image based simulation. A representative movie generated by the plugin, ISBI Challenge Track Generator, of an open platform software ICY. Seed = 123,456, SNR = 4, sequence length = 10, particle density = 1000, sigma = 10 in the particle motion with creator type “BROWNIAN_UNIFORM”. The other parameters are set to default, which means the extinction rate of each particle is 0.05. (TIFF 2561 kb) [file 12918_2018_526_MOESM4_ESM.tif]

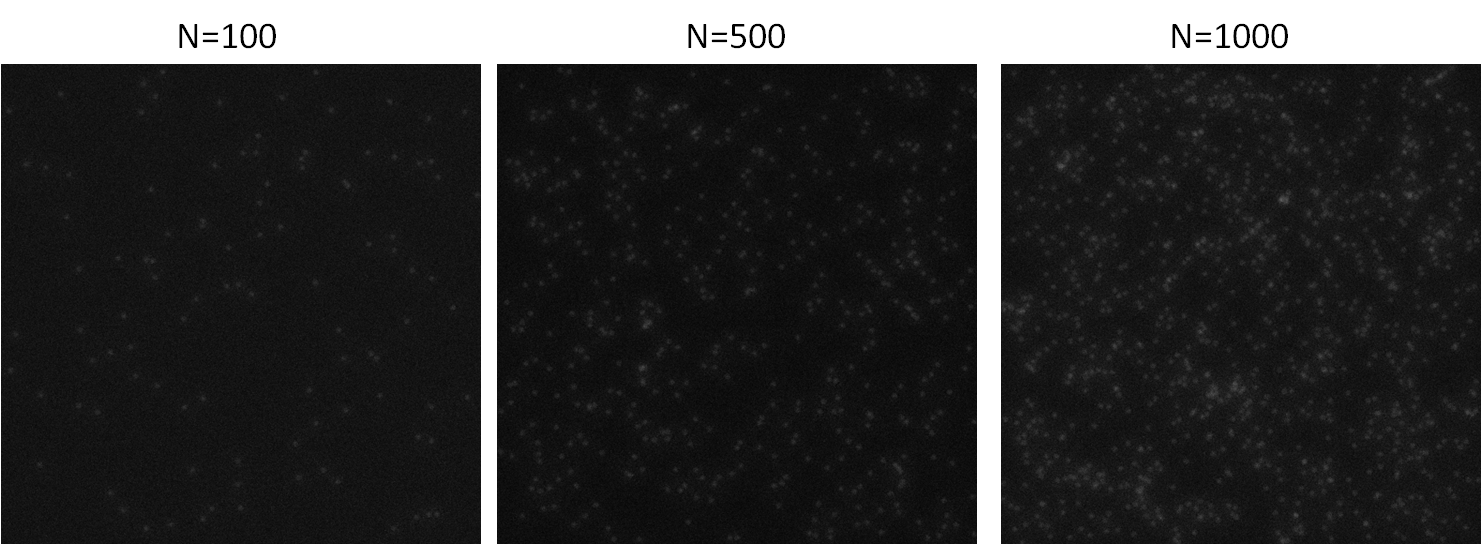

Supplement: Supplementary file 6 — Figure S4. Representative images of the image based simulation. Representative images generated by the plugin, ISBI Challenge Track Generator, of an open platform software ICY. Seed = 123,456, SNR = 4, sequence length = 10, sigma = 10 in the particle motion with creator type “BROWNIAN_UNIFORM”. Particle densities are 100, 500 and 1000, respectively. The other parameters are set to default. (PNG 517 kb) [file 12918_2018_526_MOESM5_ESM.png]
